# Antisense Oligonucleotide Induction of Progerin in Human Myogenic Cells

**DOI:** 10.1371/journal.pone.0098306

**Published:** 2014-06-03

**Authors:** Yue-Bei Luo, Chalermchai Mitrpant, Abbie M. Adams, Russell D. Johnsen, Sue Fletcher, Frank L. Mastaglia, Steve D. Wilton

**Affiliations:** 1 Centre for Neuromuscular and Neurological Disorders, Australian Neuro-Muscular Research Institute, University of Western Australia, Perth, Australia; 2 Department of Neurology, Xiangya Hospital, Central South University, Changsha, China; 3 Department of Biochemistry, Faculty of Medicine, Siriraj Hospital, Mahidol University, Bangkok, Thailand; 4 Centre for Comparative Genomics, Murdoch University, Perth, Australia; 5 Institute for Immunology & Infectious Diseases, Murdoch University, Perth, Australia; The John Curtin School of Medical Research, Australia

## Abstract

We sought to use splice-switching antisense oligonucleotides to produce a model of accelerated ageing by enhancing expression of progerin, translated from a mis-spliced lamin A gene (*LMNA*) transcript in human myogenic cells. The progerin transcript (*LMNA Δ150*) lacks the last 150 bases of exon 11, and is translated into a truncated protein associated with the severe premature ageing disease, Hutchinson-Gilford progeria syndrome (HGPS). HGPS arises from *de novo* mutations that activate a cryptic splice site in exon 11 of *LMNA* and result in progerin accumulation in tissues of mesodermal origin. Progerin has also been proposed to play a role in the ‘natural’ ageing process in tissues. We sought to test this hypothesis by producing a model of accelerated muscle ageing in human myogenic cells. A panel of splice-switching antisense oligonucleotides were designed to anneal across exon 11 of the *LMNA* pre-mRNA, and these compounds were transfected into primary human myogenic cells. RT-PCR showed that the majority of oligonucleotides were able to modify *LMNA* transcript processing. Oligonucleotides that annealed within the 150 base region of exon 11 that is missing in the progerin transcript, as well as those that targeted the normal exon 11 donor site induced the *LMNA Δ150* transcript, but most oligonucleotides also generated variable levels of *LMNA* transcript missing the entire exon 11. Upon evaluation of different oligomer chemistries, the morpholino phosphorodiamidate oligonucleotides were found to be more efficient than the equivalent sequences prepared as oligonucleotides with 2′-*O*-methyl modified bases on a phosphorothioate backbone. The morpholino oligonucleotides induced nuclear localised progerin, demonstrated by immunostaining, and morphological nuclear changes typical of HGPS cells. We show that it is possible to induce progerin expression in myogenic cells using splice-switching oligonucleotides to redirect splicing of *LMNA*. This may offer a model to investigate the role of progerin in premature muscle ageing.

## Introduction

Hutchinson-Gilford progeria syndrome (HGPS) is a rare premature ageing disease caused by mutations in *LMNA* that activate a cryptic splice site in exon 11 [1]. Induction of this inappropriate alternative splicing leads to the loss of 150 bases from the end of exon 11, and results in the translation of a truncated protein isoform, progerin. Compared with the normal translation product prelamin A, progerin lacks an endoproteolytic site and retains a farnesyl group on its carboxyl terminal. How progerin overexpression causes premature ageing is still uncertain. Accumulation of the permanently farnesylated progerin in the nuclear membrane results in abnormalities of nuclear shape, genome instability, and downstream activation of Notch and p53 pathways [2], [3]. Trace amounts of progerin have also been observed in several normal human tissues, although its biological significance and role in normal ageing remain to be determined [3]–[5].

Antisense oligonucleotides (AOs) can be designed to anneal to RNA by Watson-Crick hybridisation, and depending upon the base modifications and backbone chemistry, may exert their effects on gene expression through different mechanisms. An early application of AOs was to suppress expression of target gene and this was commonly achieved by recruitment of RNase H to degrade mRNA of a RNA: DNA oligonucleotide hybrid [6], [7]. AOs can also be used to redirect pre-mRNA processing [8], [9]. Since at least 74% of gene transcripts are alternatively spliced, splice-switching strategies could be broadly applicable to many different conditions [10]. Furthermore, it is estimated that 10-15% of pathogenic mutations affect gene splicing, although this number is now considered to be an underestimate [11], [12].

AO induced exon skipping, exon retention and abrogation of the usage of alternative splice sites have been reported to by-pass or suppress pathogenic mutations in Duchenne muscular dystrophy, spinal muscular atrophy and thalassemia, respectively [13]–[15]. Splice-switching AOs were able to mask abnormal splice sites in β-globin introns and force the aberrant splicing to default back to the normal pattern in β-thalassemia [16]. Employing the same principle, abnormal *LMNA* splicing was suppressed by a phosphorodiamidate morpholino oligonucleotide annealed to the aberrant cryptic splice site in the *LMNA* exon 11 pre-mRNA in HGPS cells [17].

Although splice-switching AOs can be used for therapeutic purposes by correcting defective gene transcripts, the same strategy can also be used to disrupt normal gene expression and induce pathological models of disease. Fong and colleagues have demonstrated the activation of the cryptic splice site activated in HGPS in normal human fibroblasts by targeting 2′-*O*-methoxy-ethyl AOs to motifs near the cryptic splice site in exon 11 [18].

Ageing in skeletal muscle is associated with loss of muscle bulk and strength, eventually resulting in significant functional disabilities. The processes responsible for muscle senescence are incompletely understood, but it is known that multiple factors play a role [19], including the accumulation of lifelong exposure to extrinsic detrimental factors like exercise damage, accumulative mitochondrial DNA mutations, increased free radicals and decreased oxidative response, reduced protein turnover capacity, low-grade systemic inflammation, and impaired neuromuscular junction function [20]–[23]. Nevertheless, there are few models that specifically address muscle ageing [24], [25]. In another study [26], we observed low-level accumulation of progerin in normal human skeletal muscle, but it is unclear if the levels detected are sufficient to play a role in the ageing process.

Here we report the use of two different types of splice-switching AOs to redirect processing of exon 11 of *LMNA*, so as to enhance expression of the progerin isoform in human myogenic cells and generate an *in vitro* model of premature muscle ageing.

## Experimental Procedures

### Antisense oligonucleotides

2′-*O*-methyl modified bases on a phosphorothioate backbone (2OMe AOs) were synthesised in-house on an Expedite 8909 Nucleic Acid Synthesiser (Applied Biosystems, Framingham, MA) using the 1 µmol thioate synthesis protocol. Phosphorodiamidate morpholino oligonucleotides (PMOs) were obtained from GeneTools, LLC (Philomath, OR).

Nomenclature of AOs adopted the method described by Mann et al [27]: species (‘H’ for homo sapiens), exon number, acceptor (A)/donor (D) site, coordinate (‘+’ for exon, ‘−’ for intron).

### Tissue samples

Surplus material from de-identified *vastus lateralis* muscle biopsies, obtained from individuals undergoing screening for malignant hyperthermia (MH) was provided by the Department of Pathology, Royal Perth Hospital, with informed consent. These individuals were found to be MH-negative based upon *in vitro* contracture testing, and had normal muscle histology. Additional muscle tissues and skin tissues from healthy individuals were obtained after informed consent and stored at −80°C. All procedures were approved by the Royal Perth Human Ethics Committee (reference number: 2006-073).

### Cell culture and AO transfection

Primary human myogenic cells were prepared and differentiated as described previously [28]. Human cells were transfected with 2OMe AOs complexed with Lipofectamine 2000 (Invitrogen, Melbourne, Australia): 2OMe at 1∶1 (w∶w) ratio.

Human myogenic cells were transfected with PMOs using the Amaxa Nucelofector electrophoration system (Lonza, Basel, Switzerland) with P3 primary cell 4D-Nucleofector X kit and pulsed with the programme CM-138 according to the manufacturer's instructions.

### Reverse-transcriptase polymerase chain reaction (RT-PCR)

RNA was extracted from cells 48 hr (2OMe AOs) or 72 hr (PMOs) after transfection using Trizol (Invitrogen) according to manufacturer's instructions. One-step RT-PCR was undertaken essentially as described previously [26]. Briefly, samples were incubated at 75°C for 30 minutes for reverse transcription step, followed by 3 minutes incubation at 94°C to denature the templates, followed by 30 cycles of PCR (denaturation at 94°C for 30 seconds, annealing at 55°C for 1 minute and extension at 72°C for 2 minutes). Amplification primers were: LAf (exon 9/10 junction), 5′-ATCAACTCCACTGGGGAAGAAGT-3′, LAr (exon 12) 5′-ATGTGGAGTTTCCTGGAAGCAG-3′; LCf (exon 6), 5′-GAGCGGGAGATGGGAGAT-3′, LCr (exon 10) 5′-TCAGCGGCGGCACCACTCA-3′). Amplification products were separated on 2% agarose gel and images captured using a Chemi-smart 3000 system (Vilber Lourmat, Marne-la-Vallée, France). The identity of the PCR amplicons were confirmed by direct DNA sequencing.

### Western blotting

Three hundred and sixty thousand human myogenic cells were seeded into T25 flasks and incubated for 48 hr before transfection with AOs as described. Forty-eight hr after transfection, cells were harvested from the wells and centrifuged at 14,000 rcf for 3 minutes to collect the cell pellets. Approximately 4.5 mg of cells were lysed with 100 µl of 125 mM Tris-HCl (pH 6.8), 15% SDS (w∶v), 10% glycerol (v∶v), 0.5 mM phenylmethylsulfonyl fluoride and 9 µl protease inhibitor cocktail (Sigma Aldrich, Sydney, Australia). Western blots were carried out essentially as described by Cooper [29]. Briefly, 4 µl aliquots of protein extract were separated on NuPAGE 4–12% Bis-Tris gels (Life Technologies, Mulgrave, Australia) and stained with 0.2% Coomassie blue and destained with 0.7% acetic acid. Gel densitometry was used to estimate relative myosin expression to ensure equal protein loading on subsequent gels for western blotting. Protein extracts were fractionated on NuPAGE 4–12% Bis-Tris gels (Life Technologies) and electro-transferred to polyvinylidene fluoride membrane (Pall, Melbourne, Australia). The membranes were incubated with primary antibodies (anti-lamin A/C, Millipore, Kilsyth, Australia, 1∶100; anti-dysferlin, Leica Microsystems, North Ryde, Australia, 1∶1,500) overnight and then labeled with anti-mouse secondary antibody (Novex Western Breeze Immunodetection kit, Life Technologies) for 1 hr. After incubation with Chemiluminescent substrate for 5 min, images were captured by a Chemi-Smart 3000 gel documentation system (Vilber Lourmat) using Chemi-capt software with image analysis performed using Bio-1D software.

### Confocal microscopy

After PMO or 2OMe transfection, 180,000 myogenic cells were placed in a glass bottom petri dish (MatTek, Ashland, MA) and cultured in 5% horse serum in Dulbecco's modified Eagle medium for 72 hr before immunostaining. Cultures were incubated with anti-progerin (Abcam, Sapphire Bioscience, Waterloo, Australia) or lamin A/C (Millipore) antibody for 2 hr and followed by incubation with Alexor Fluor 488 goat anti mouse immunoglobulin (Invitrogen, 1∶400) for 1 hr at room temperature, and then counterstained with Hoechst 33342 (Sigma Aldrich, 1∶4,000) for 5 min. After rinsing with PBS, slides were viewed under a Nikon A1Si laser scanning confocal microscope (Coherent Scientific, Hilton, Australia).

## Results

### AO induction of LMNA Δ150 and LMNA ΔE11 transcripts

**Figure 1:**
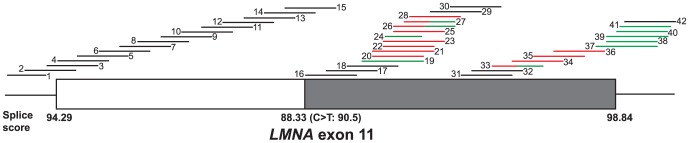
Schematic of *LMNA* exon 11 and annealing AOs. The grey bar represents the 150 bases omitted from the *LMNA Δ150* transcript. The AOs assessed in this study are shown according to their coordinates on exon 11. AOs that have minimal splicing modulatory effect are shown in black, AOs inducing predominantly cryptic splicing activation in red, AOs inducing mainly exon 11 skipping in green. Splicing strength scores are calculated by Human Splice Finder (http://www.umd.be/HSF/).

**Figure 2:**
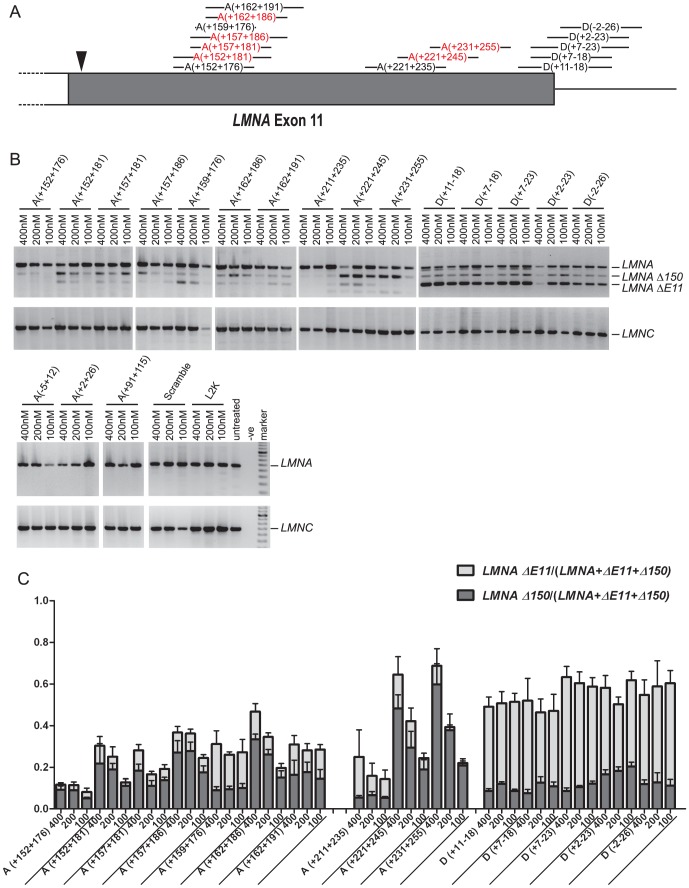
RT-PCR showing changes in *LMNA* splicing after transfecting with 2OMe AOs. (A) AO annealing location within the 150 base region of exon 11excluded in HPGS (in grey). The arrowhead denotes the site of the classic HGPS C>T mutation. AOs that induce the greatest degree of cryptic splicing activation are shown in red. (B) Representative gel images of RT-PCR *LMNA*-related products from cells transfected over a range of concentrations. A smaller fourth *LMNA* transcript product induced in cells transfected with 11A(+211+235) to 11A(+231+255) was identified as missing exons 10+11. (C) Semi quantitative analysis by densitometry of gel band intensity, indicating levels of different *LMNA* transcripts. Bars denote mean ±SE.

Forty-two 2OMeAOs, 18–30 bases in length, were designed to target the *LMNA* pre-mRNA sequence between the end of intron 10 and the beginning of intron 11 (Figure 1, Table 1). AOs targeting the pre-mRNA from 30 bases downstream of the cryptic splice site to the donor site, were able to induce some *LMNA Δ150* transcript production, as assessed by RT-PCR (Figure 2). In addition to the *LMNA Δ150* transcripts, there were also variable levels of exon 11 skipping (*LMNA ΔE11*), particularly with AOs annealing close to the donor site. Cryptic splice site activation and exon 11 skipping was generally stronger when AOs were targeted to the area near the donor site than the domain 30 bases downstream to the cryptic splice site. AOs 11A(+152+181), 11A(+157+186) and 11A(+162+186) were the most efficient *LMNA Δ150*-inducing AOs targeting the domain 30–70 bases downstream of the HGPS splice site (Figure 2B,C). The AOs 11A(+221+245) and 11A(+231+255) annealed upstream of the wild-type donor site, and induced the highest level of *LMNA Δ150* induction of all the 2OMe AOs tested (Figure 2B,C). Transfection of AOs that anneal to the acceptor site or the first 120 bases upstream of the cryptic splice site did not have any obvious effect on the splicing of *LMNA* exon 11 (Figure 2B). The identities of the *LMNA Δ150* and *LMNA ΔE11* transcripts were confirmed by direct DNA sequencing.

**Table 1:** Antisense oligonucleotides tested in the present study.

Number	Nomenclature and Coordinates	Sequence (5′-3′)	GC content
1	HLmnA11A (−5−23)	aag gga gac aag acu cag g	52.63%
2	HLmnA11A (−15+10)	agu ggg agc ccu ggg aag gga gac a	60.00%
3	HLmnA11A (−5+20)	gag cug cug cag ugg gag ccc ugg g	72.00%
4	HLmnA11A (+2+26)	ucc ccc gag cug cug cag ugg gag c	72.00%
5	HLmnA11A (+11+35)	uca gcg ggg ucc ccc gag cug cug c	76.00%
6	HLmnA11A (+21+45)	cag guu gua cuc agc ggg guc ccc c	72.00%
7	HLmnA11A (+31+55)	ugc gcg agc gca ggu ugu acu cag c	64.00%
8	HLmnA11A (+41+65)	cac agc acg gug cgc gag cgc agg u	72.00%
9	HLmnA11A (+51+75)	gca ggu ccc gca cag cac ggu gcg c	76.00%
10	HLmnA11A (+61+85)	cag gcu gcc cgc agg ucc cgc aca g	79.17%
11	HLmnA11A (+71+95)	gcc uug ucg gca ggc ugc ccg cag g	76.00%
12	HLmnA11A (+81+105)	gcu ggc aga ugc cuu guc ggc agg c	68.00%
13	HLmnA11A (+91+115)	cuc cug agc cgc ugg cag aug ccu u	64.00%
14	HLmnA11A (+101+125)	ccc acc ugg gcu ccu gag ccg cug g	76.00%
15	HLmnA11A (+111+135)	gau ggg ucc gcc cac cug ggc ucc u	72.00%
16	HLmnA11A (+121+145)	agc cag agg aga ugg guc cgc cca c	68.00%
17	HLmnA11A (+131+155)	gag gca gaa gag cca gag gag aug g	60.00%
18	HLmnA11A (+141+165)	cgu gac acu gga ggc aga aga gcc a	60.00%
19	HLmnA11A (+147+176)	cug cga gug acc gug aca cug gag gca gaa	60.00%
20	HLmnA11A (+152+176)	cug cga gug acc gug aca cug gag g	64.00%
21	HLmnA11A (+152+181)	ggu agc ugc gag uga ccg uga cac ugg agg	63.33%
22	HLmnA11A (+157+181)	ggu agc ugc gag uga ccg uga cac u	60.00%
23	HLmnA11A (+157+186)	acu gcg gua gcu gcg agu gac cgu gac acu	60.00%
24	HLmnA11A (+159+176)	cug cga gug acc gug aca	61.11%
25	HLmnA11A (+162+186)	acu gcg gua gcu gcg agu gac cgu g	64.00%
26	HLmnA11A (+162+191)	ccc aca cug cgg uag cug cga gug acc gug	66.67%
27	HLmnA11A (+167+191)	ccc aca cug cgg uag cug cga gug a	64.00%
28	HLmnA11A (+171+195)	gcc ccc cac acu gcg gua gcu gcg a	72.00%
29	HLmnA11A (+181+205)	cac ccc cac ugc ccc cca cac ugc g	76.00%
30	HLmnA11A (+191+215)	ccg aag cug cca ccc cca cug ccc c	76.00%
31	HLmnA11A (+196+220)	ugu ccc cga agc ugc cac ccc cac u	68.00%
32	HLmnA11A (+201+225)	cag auu guc ccc gaa gcu gcc acc c	64.00%
33	HLmnA11A (+211+235)	agc ggg uga cca gau ugu ccc cga a	60.00%
34	HLmnA11A (+221+245)	agg agg uag gag cgg gug acc aga u	60.00%
35	HLmnA11A (+231+255)	gga guu gcc cag gag gua gga gcg g	68.00%
36	HLmnA11A (+241+265)	uuc ggg ggc ugg agu ugc cca gga g	68.00%
37	HLmnA11D (+11−18)	aaa gca gag aca acu cac cug ggu ucg gg	55.17%
38	HLmnA11D (+7−18)	aaa gca gag aca acu cac cug ggu u	48.00%
39	HLmnA11D (+7−23)	gag aca aag cag aga caa cuc acc ugg guu	50.00%
40	HLmnA11D (+2−23)	gag aca aag cag aga caa cuc acc u	48.00%
41	HLmnA11D (−2−26)	uug gag aca aag cag aga caa cuc a	44.00%
42	HLmnA11D (−5−29)	gau uug gag aca aag cag aga caa c	44.00%

Further refinement of AOs that induced the most pronounced induction of the *LMNA Δ150* transcript was undertaken. Lengthening the AO, 11A(+157+181), by five bases at the 3′ end (11A(+157+186)) increased cryptic splicing, whereas removing bases from each end (11A(159+176)) or moving the annealing coordinates 5 bases downstream, as well as extending the 3′ end again (11A(+162+191)) resulted in less splice switching activity (Figure 2C). Moving 11A(+211+235) 10 bases further toward the donor site (11A(+221+245)) or 20 bases further (11A(+231+255)) dramatically increased cryptic splicing (Figure 2C).

**Figure 3:**
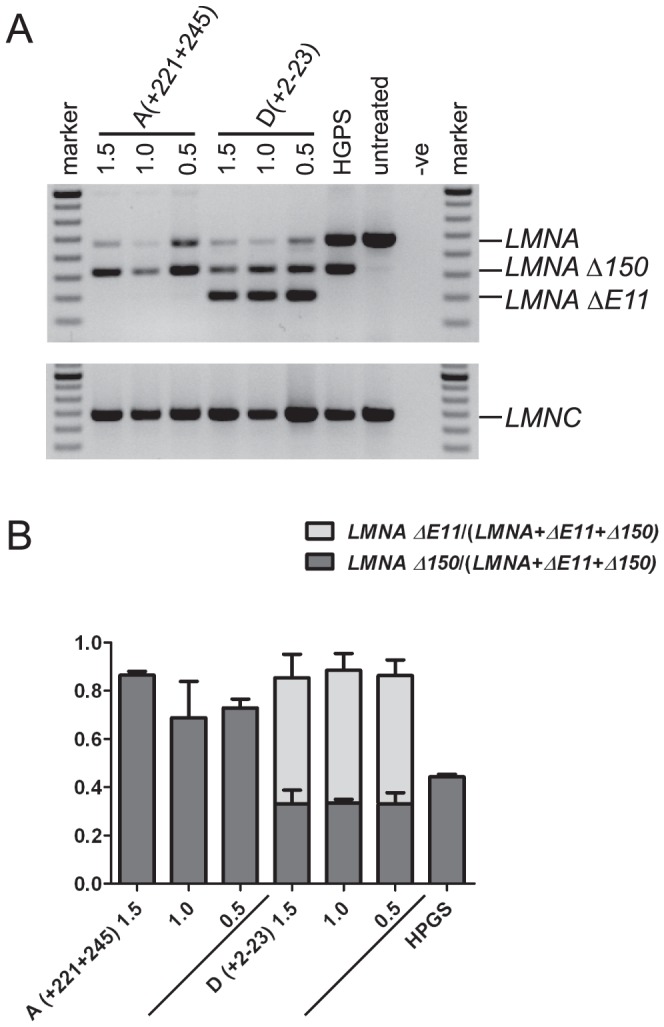
RT-PCR showing *LMNA Δ150* induction after transfecting with PMOs. (A) PMO 11A(+221+245) only induces *LMNA Δ150* (537 bp product) whereas 11D(+2−23) promotes both alternative splicing and exon skipping (417 bp). (B) Bar chart shows amplicon band intensity (mean±SE). AO concentrations are in µM.

Two 2OMe AOs, shown to effectively modify *LMNA* splicing were selected for further evaluation after being synthesised as PMOs: 11A(+221+245) was selected since *LMNA Δ150* induction was greater than exon 11 skipping, whereas 11D(+2−23) induced robust exon 11 skipping with reduced *LMNA Δ150* generation (Figure 3). Compared with its 2OMe equivalent, the PMO 11A(+221+245) appeared more specific in terms of cryptic splicing site activation. Both PMOs induced higher levels of *LMNA Δ150* than their 2OMe counterparts (Figure 2,3, 11A(+221+245) PMO 80.2% vs 2OMe 44.7%, 11D(+2−23) PMO 33.7% vs 2OMe 18.4%). The level of *LMNA Δ150* was even higher in myogenic cells treated with 11A(+221+245) PMO than in HGPS fibroblast cultures (Figure 3).

### Progerin induction in PMO transfected myogenic cells

**Figure 4:**
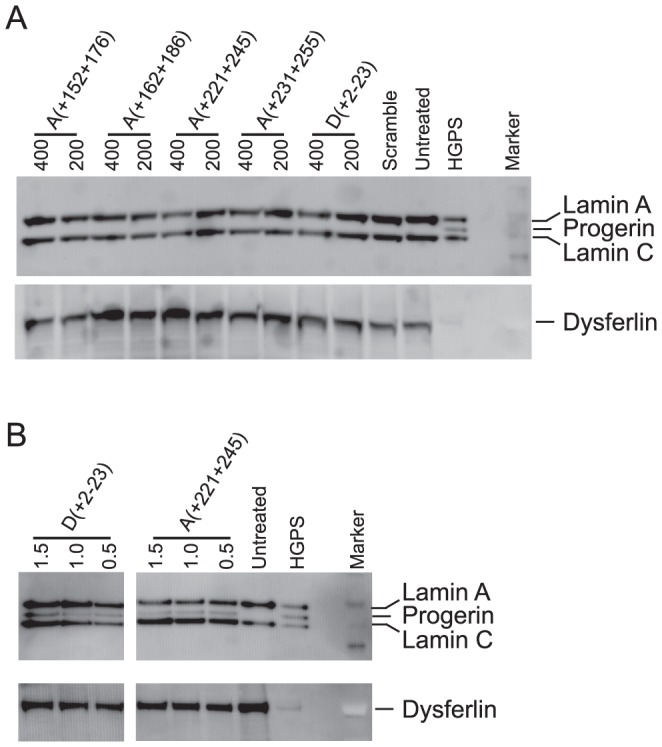
Western blotting demonstrating the inability to detect progerin in cells after transfecting with 2OMe AOs (A) and progerin production after PMO transfection (B). AO concentrations are in nM in (A) and µM in (B).

Despite inducing robust expression of the *LMNA Δ150* transcript, the western blots of extracts from 2OMe AO transfected cells demonstrated only wild-type lamin A and C bands, with no detectable progerin (Figure 4A). In contrast, both PMOs induced sufficient splice-switching to generate detectable levels of progerin (Figure 4B). Theoretically, the lamin A ΔE11 protein should go through the first three steps of post-translational processing, and since it is only one amino acid smaller than lamin C, it is not distinguishable from lamin C using our current protein detection system.

### Accumulation of progerin induces abnormalities in nuclear shape

**Figure 5:**
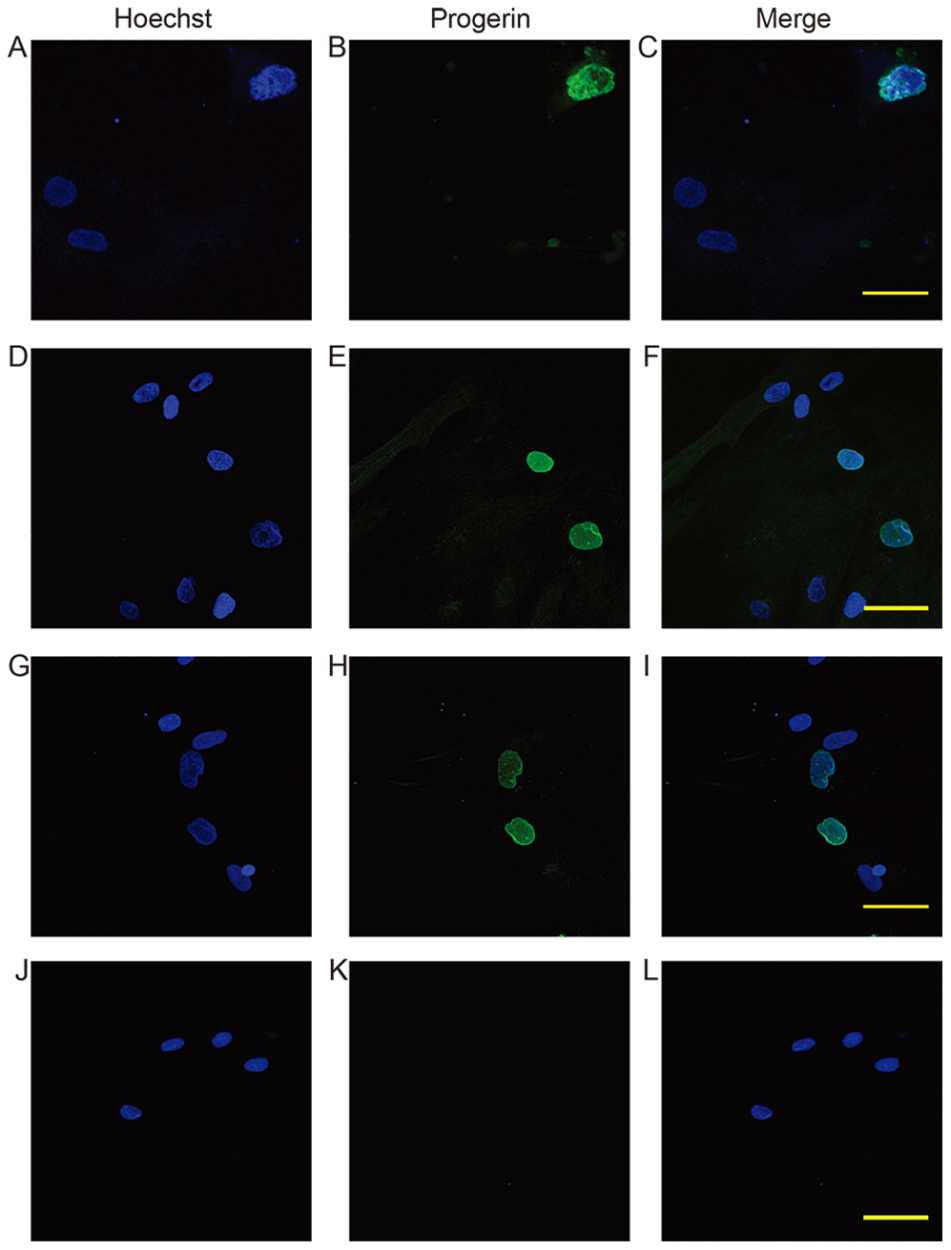
Confocal fluorescence microscopy with false colour showing the localization of progerin (green) in nuclei (blue) in human myogenic cells. In HGPS fibroblast cultures, progerin positive nuclei are mostly lobulated or trabeculated (A–C). Human myogenic cells transfected with PMOs also demonstrated abnormally shaped progerin reactive nuclei (D–F: transfected with 1 µM PMO 421; G–I: 0.5 µM PMO 422). Untreated cells did not contain any detectable progerin positive nuclei (J–L). Magnification: 60×. Scale bar: 50 µm.

PMO-treated myogenic cells and HGPS fibroblasts were stained with a progerin-specific antibody to assess its distribution. In HGPS fibroblast cultures, 25.2% (115/456) of nuclei were immuno-reactive for progerin. In human myoblast cultures, consistent with the RT-PCR results, cells transfected with the PMO 11D(+2−23) at 0.5 and 1 µM concentration induced 11.2% (71/632) and 15.2% (247/1625) progerin positive nuclei, whereas 11A(+221+245) induced marginally more positive nuclei (13.2% (93/705) and 17.1% (114/667)) respectively). Nuclei from the PMO-treated cells that stained positive for progerin generally demonstrated abnormal shapes (e.g. lobulation and pouching) similar to those of HGPS nuclei, and some contained progerin aggregates (Figure 5A–I). Progerin-positive nuclei were not found in untreated human myogenic cells (0/541, Figure 5J–L) or cells transfected with 2OMe AOs (data not shown).

Cells were labelled with anti-lamin A/C antibody to evaluate nuclear shape abnormalities. There were 8.97% (14/156) and 11.80% (42/356) abnormally shaped nuclei in cells nucleofected with PMO11D(+2−23) at 0.5 and 1 µM concentration respectively, while 5.37%(18/335) and 5.99%(10/167) in cells transfected with 2OMe11D(+2−23) at 0.5 and 1 µM concentration. In comparison, the percentage of aberrant nuclei in cells nucleofected with 0.5 and 1 µM PMO11A(+221+245) was 5.74% (7/122) and 8.12% (19/234) respectively, whereas that in cells transfected with 0.5 and 1 µM 2OMe11A(+221+245) was 4.67% (7/150) and 6.29% (21/334). Cells transfected with 0.5 and 1 µM scrambled 2OMe AO 8.9–11.7 also demonstrated 5.13% (4/98) and 5.32% (5/94) aberrant nuclei, and untreated cells 2.13% (2/93).

## Discussion

Under normal conditions, alternative splicing of *LMNA* gives rise to at least three different isoforms, lamin A, C and lamin A Δ10 [30], [31]. The predominant isoforms, lamin A and C, are involved in a myriad of physiological processes, including maintaining nuclear shape, DNA replication and transcription, and enabling interaction between nucleoplasm and cytoplasm by connecting the nucleo- with the cyto-skeleton of the cell [32]–[35]. It is therefore not surprising that in HGPS, aberrant splicing arising from activation of a cryptic splice site and production of the progerin isoform lead to a wide range of downstream events culminating in premature cellular senescence [2], [36], [37]. *LMNA* mutations have been associated with several clinically distinct neuromuscular disorders including Emery-Dreifuss muscular dystrophy, limb girdle muscular dystrophy type 1B and Charcot-Marie-Tooth diseases type 2B1 [38]–[40]. Lamin A/C expression is also important in muscle differentiation and maintenance of muscle function [41]–[43]. We have demonstrated the presence of progerin in normal skeletal muscles [26] and other researchers have reported detecting progerin in other normal tissues including blood vessels, skin, liver and heart [3], [4], [44]. By using splice-switching AOs, we show here that progerin-overexpressing myonuclei exhibit aberrant shapes similar to those in HGPS cells, and to nuclei in normal ageing cells [3], that may be a relevant *in vitro* model of accelerated muscle ageing.

The AOs annealing to motifs across exon 11 of *LMNA* pre-mRNA could be divided into 3 classes according to their effects on *LMNA* splicing: 1- those that exerted no or minimal effects on *LMNA* pre-mRNA processing, 2- those that induced primarily exon 11 skipping, and progerin production to a lesser extent, and 3- those that promoted selection and usage of the cryptic splice site leading to the production of the truncated lamin A isoform, progerin with some exon 11 skipping.

Our experience with the design of splice-switching AOs to induce exon skipping in the dystrophin gene transcript is that the donor sites are generally unresponsive splice switching targets for the majority of constitutively expressed exons. On the other hand, the dystrophin acceptor sites and the first half of exons have proved to be more amenable targets for exon skipping [45]. Directing AOs to mask either donor or acceptor splice sites, both crucial motifs in the splicing process, is not guaranteed to identify a compound capable of modifying processing of the target transcript. In direct contrast to our previous studies on dystrophin, the acceptor site and first half of *LMNA* exon 11 were unresponsive to AO splice modulation, while AOs targeting the latter half of *LMNA* exon 11 and the donor splice site did modify processing of the transcript. There may be restricted access to the *LMNA* exon 11 acceptor site because of secondary RNA conformation or an enrichment of proteins binding in this domain that prevent oligonucleotide binding. By masking the latter half of *LMNA* exon 11 and the constitutive donor splice site, the splicing machinery either fails to recognise the entire exon or is forced to use the cryptic splice site of exon 11 activated in HGPS.

Most of the AOs found to influence *LMNA* splicing induced a mixture of transcripts, some missing exon 11 and others missing the 150 bases downstream of the cryptic splice site. This implies two mechanisms, either enhancing recognition of the cryptic splice site, or blocking selection of the entire exon and inducing its loss from the mature mRNA. Some AOs may influence exonic splicing enhancer (ESE) and/or an exonic splicing silencer (ESS) and direct the splicing machinery to use the cryptic splice site or mask the entire exon. The GC content of AOs targeting this area are similar (Table 1), therefore it is unlikely that the annealing capacity of these AOs plays a significant role in the different levels of alternative splicing. Instead, the results suggest that the motifs targeted by 11A(+177+186) (57 bases downstream to the cryptic splice site) and 11A(+236+255) (116 bases downstream to the cryptic splice site, 15 bases upstream to the donor site) may act as ESEs for the consensus donor site or ESSs for the cryptic splice site. A previous study by Lopez-Mejia and colleagues demonstrated that the exon 11 cryptic splice site is engaged in a stem-loop like structure of the pre-mRNA, which limits its accessibility by the spliceosome [46]. The HGPS C>T mutation potentially opens up the loop structure and facilitates recognition of the cryptic splice site by the splicing machinery. This study also proposed that the region 50 to 66 bases downstream of the cryptic splice site is in a single-stranded region and is likely to be highly accessible to splicing factors, as well as to the AOs. Oligonucleotides targeting this area may have higher affinity for the pre-mRNA and cause more dramatic effects on *LMNA* splicing.

Redirection of *LMNA* pre-mRNA splicing was induced with two different splice-switching oligonucleotide chemistries, 2OMe AOs and PMOs. Although some of the 2OMe AOs induced robust progerin mRNA production, it was always associated with variable levels of *LMNA* exon 11 excision. Nevertheless, despite 2OMe AOs inducing the Δ150 progerin mRNA, as assessed by RT-PCR, it was not possible to detect progerin protein in these cells by western blotting. In contrast, the same sequences synthesised as PMOs were able to induce specific and efficient cryptic splice activation that resulted in readily detectable levels of progerin, as well as morphological nuclear changes resembling those that occur in HGPS. This difference in transfection outcome between the two oligomer chemistries is consistent with our findings in Duchenne muscular dystrophy models and exon skipping. The PMOs are more effective *in vitro* and *in vivo* than their 2OMe counterparts [47]–[49]. Heemskerk and colleagues also demonstrated that PMOs could induce 9 to 10 fold more dystrophin in the *mdx* mouse than the equivalent 2OMeAOs administered at the same dose [50].

This is the first time we have demonstrated the greater splice switching potency of the PMOs in changing the splicing pattern and protein production of a gene other than dystrophin. This may indicate a fundamental limitation of the 2OMe AOs as clinical splice switching compounds. Recently, a DMD exon skipping trial using a 2OMeAO was halted as primary and secondary endpoints were not met. While disappointing for the DMD community, these trial results cannot be regarded as surprising as there had been no unequivocal increases in dystrophin after 2OMe AO treatment. In contrast, another DMD exon skipping trial using an oligomer composed of the PMO chemistry appears to have stabilized ambulation in 10 out or 12 trial participants, with robust dystrophin being detected in muscle biopsies from these boys [51].

A previous study by Fong and colleagues employed another splice switching oligonucleotide chemistry, 2′-methoxy-ethyl modified bases on a phosphorothioate backbone (2′-MOE) to activate the cryptic splice site in normal human fibroblasts [18]. Their most effective AO targeted 34 to 56 bases downstream of the HGPS cryptic splice site, whereas in this study two other domains downstream to the cryptic splice site (57 to 66 bases, and 116 to 135 bases) were most efficient in inducing progerin. Another difference between this study and that by Fong et al, is that our study identified a wider area that can mediate progerin expression (from 50 bases downstream of the cryptic splice site of exon 11 to the beginning of intron 11). Further, whereas a seemingly precise switching from lamin A to progerin production was achieved by Fong et al, variable degrees of exon 11 skipping invariably accompanied increased utilization of the cryptic site in our study with 2OMe AOs. For example, the 2OMe AO 11A(+159+176) has the same sequence as one of the most efficient AOs (324) described by Fong et al., and caused both cryptic splice site activation and exon 11 skipping in our study. Several factors may contribute to the discrepancies between the two studies, including the use of different cell strains (i.e. fibroblasts vs myogenic cells) and different AO chemistries (2′-MOE vs 2OMe). However, we also transfected normal human skin fibroblasts with our AOs and the resulting splicing pattern (ie the mixed induction of *LMNA Δ150* and *LMNA ΔE11*, *LMNA Δ150*/*LMNA*, *LMNA ΔE11*/*LMNA* ratios) was identical to that induced in myogenic cells (data not shown). It is therefore unlikely that splicing environment in different tissues is responsible for the disparity in splicing redirection in the different studies [46], [52]. The variable efficiencies with which progerin was induced by our 2OMe AOs and PMOs also support the possibility that the oligonucleotide chemistry has a major impact on transfection outcomes. But other factors may also contribute: different AO length (25–30 mer vs 16–20 mer), transfection concentrations (100–400 nM vs 2.5–100 nM) and PCR amplification conditions.

We could induce the accumulation of progerin as well as lamin A ΔE11 in human myogenic cells using splicing switching AOs. Both progerin and lamin A ΔE11 lack a proteolytic site for post-translational modification of the precursor protein prelamin A. Consequently, both aberrant proteins retain a farnesyl group at the C terminal, which is normally cleaved from the wild-type mature lamin A. It is proposed that the farnesyl group plays a key role in the pathogenesis of farnesylated prelamin A-accumulating diseases [53], [54]. The retention of the farnesyl group prevents the progerin from disassociating from the nuclear lamina during the cell cycle and disrupts mitosis [36].

Accumulation of lamin A ΔE11 causes another fatal progeroid disease, restrictive dermopathy [55]. To date there are few studies regarding the pathophysiology of lamin A ΔE11, hence the splice-switching method here may offer an inducible model to further study this disease. Given that the lamin A ΔE11 product, like progerin, is presumably permanently farnesylated and that restrictive dermopathy demonstrates similar nuclear abnormalities to HGPS, it is possible that lamin A ΔE11 will have similar downstream effects to those caused by progerin. Lamin A ΔE11 is probably as deleterious as, if not more so, progerin in HGPS, considering the extreme phenotype of restrictive dermopathy. Indeed, the fact that accumulation of progerin and lamin A ΔE11 can both cause restrictive dermopathy suggests that HGPS and restrictive dermopathy belong to the same clinical spectrum of diseases caused by farnesylated prelamin A [56]. Therefore, although there is a mixture of cryptic splicing activation and exon 11 skipping in the AO treated myogenic cells in the present study, it is our belief that the induced products, progerin and lamin A ΔE11, exert similar effects in cells to cause accelerated ageing. Consistent with this hypothesis, similarly mis-shapen myonuclei were found in myogenic cells treated with the PMOs that induced progerin alone and both progerin and lamin A ΔE11.

Premature ageing can be induced in fibroblasts and human midbrain dopamine neurons derived from induced pluripotent stem (iPS) cells by transfection with a synthetic RNA that encodes progerin tagged with GFP [57]. Enhanced expression of progerin was only achieved after 3 and 5 repeats of daily transfection in iPS-fibroblasts and iPS-neurons respectively. In contrast, the splice switching PMOs in this study induced more readily detectable amounts of progerin 36 hours after transfection. It will be interesting to evaluate the consequences of progerin expression arising from PMO induced splice switching in iPS-fibroblasts and iPS-neurons.

In conclusion, we have shown that AOs targeting the putative ESEs/ESSs within exon 11 of *LMNA* or the donor site, can be used to redirect splicing in human myogenic cells, and lead to the production of two distinctive, yet functionally similar, farnesylated prelamin A isoforms (progerin and lamin AΔE11). The PMO chemistry was found to be more effective than the 2OMe chemistry in terms of specificity and progerin production. The PMOs increased production of progerin and induced the nuclear changes associated with premature ageing, similar to those that occur in HGPS. AOs therefore have the potential to manipulate splicing and induce pathogenic splicing, and changes of premature ageing in cells *in vitro*. PMO 11D(+2−23) leads to predominant exon 11 skipping and may serve as a suitable model to study the pathophysiology of lamin A ΔE11.
